# Mantels and Grates

**Published:** 1885-04

**Authors:** 


### MANTELS AND GRATES.

An open fire is the soul of the room ; it gives it vitality, life ; it draws the household around it in pleasant, cosy chat. As the body without the soul is dead, so the room without the fire is dead also. Setting aside for the moment the mere utility of the open fire as a means of ventilation and purification, let us remember that it has its spiritual, its poetical, its æsthetic side which is welcome to all men. As the soul is beautiful when shining out from a beautiful face, so a fire is more beautiful shining out from beautiful surroundings, and the grate and the mantel become important features in the room. Marble mantels have their beauty and appropriateness in certain places, as, for instance, in vast halls, where grand architectural effect is desirable ; but, to our taste, not in a home, not in quiet domestic architecture, not in the family room where the household gathers in the ordinary daily life.

Wood mantels have all the qualities that household use and beauty demand. What a wide range they offer in color, so that all taste can be suited and all styles of decoration find a harmony? We may choose the delicate purity of whitewood, or the golden glow of pine, the fine red of cherry, or purple amaranth, or the rich grains of oak and ash, or the glow and splendor of mahogany, or the dusky gleam of black walnut, or the fine soft finish of birch, or we can bring into our service unnumbered foreign woods to make beautiful the fire-place, and set a fitting frame around our beloved fire.

And just here let one thing, and a very important one be said: If you put wood into the fire it will burn! Many of our designers of wood mantels forget this, and their florid efforts blister and burn, to the despair and vexation of the carefnl housekeeper. The mantel shelf on its under side becomes a mass of blisters; the columned or bracketed sides char and blacken, and the open fire, that should give only pleasure, is regarded with anxiety, lest it shonld have in it the terrors of a conflagration. Now all of this can be easily avoided. Keep your woodwork within the line of safety. This line of safety is easily determined. We give the result of long years of experience in mantel and grate setting, of a scientific, thoughtful man, and if this rule be followed, it will result in entire safety to the most delicate wood mantel so far as burning is concerned. The rule is as follows: From the front line of your fire draw a line at an angle of about fifteen degrees, and keep all your woodwork behind that line. This is for the sides. For the top draw from the top of the fire-place opening a line at the same angle, fifteen degrees for a foot above the opening, and then change the angle to thirty-five degrees, and keep the mantel shelf and all the top wood behind this line, and you will have no more charred and blistered wood, no matter how hot the fire.

In wood mantels every taste may be gratified, the field for design is infinite. The Gothic, the Renaissance, the Colonial, the Queen Anne offer themselves in all their thousand modifications and combinations to suit modern convenience. A half century ago all our houses had wood mantels; it is no new fashion. The Colonial mantels were all brought from England, as in fact were all the materials for the house. Many of these houses may still be seen in Salem, in Marblehead, in Plymouth, and in many other old towns of New England. A friend thus describes to us some old mantels which would be the delight of art-lovers of to-day: "Wooden mantels are no new things. There were plenty before marble was thought of, and were of elaborate patterns. Some fifteen years ago I was present at the demolition of an old house in Maryland, built when Anne was queen of England. It contained two mantels of English oak, one at either end of the large parlor, with enormous fire-places, the mantels covering the entire end of the room, with cabinets, and shelves and cosy nooks, and corners without stint. The pilasters at the lower parts were worn away by the attrition of several generations of shovels, tongs, and pokers which had leaned against them, but otherwise the mantels were in perfect preservation, with joints as good as new. They had a multitude of small, deeply moulded panels, the product of Anne's time, and the wood was nearly as hard as iron.

"The facings were of large black brick, and no trace of fire was visible in any part of the woodwork, although it had been in use 160 years. The artisans of those times put their lives into a piece of work with far more persistency than is done now-a-days. It must have taken a workman an entire year to make one of these mantels, unassisted by modern machinery." In those days the chimney stack was in the centre of the house, often a vast structure in itself, a tower around which the house was built. The fire-places were vast caverns ; a tall man could walk into them without bending his head, and they were twelve feet or more in width. It was an old saying that there was space enough for an ox-cart and a yoke of oxen to be driven up the chimney. In some of the old colonial houses still standing in Marblehead and Salem, Mass., there are wood mantels covering the whole side of the room, with delicate mouldings worked in the solid wood, not glued on in the fashion of to-day, the panels enriched with carving of flowers and foliage in the very best style of the art."

To-day comfort has banished the vast cavernous fire-places, but the open fire is just as cheerful as of old, in its more modest surrounding. A half century ago there was a raid made on the old-fashioned wood mantels, and with all their quaint carving and delicate mouldings and panel-work, they were swept out for kindling wood, and everybody set up in his house the black and gold Egyptian marble mantel. But the reaction has come with an increased civilization, and wood re-asserts itself. Wood mantels, tile facings, and tile hearths are now the fashion, and a beautiful fashion it is. Long may it last.—*California Architect.*

